# Comparison of expression, purification and characterization of a new pectate lyase from *Phytophthora capsici *using two different methods

**DOI:** 10.1186/1472-6750-11-32

**Published:** 2011-04-06

**Authors:** Huizheng Wang, Li Fu, Xiuguo Zhang

**Affiliations:** 1 Department of Plant Pathology, Shandong Agricultural University, Tai'an, 271018, Shandong, China

## Abstract

**Background:**

Pectate lyases (PELs) play an important role in the infection process of plant pathogens and also have a commercial significance in industrial applications. Most of the PELs were expressed as soluble recombinant proteins, while a few recombinant proteins were insoluble. The production of a large-scale soluble recombinant PEL would allow not only a more detailed structural and functional characterization of this enzyme but also may have important applications in the food industry.

**Results:**

We cloned a new pectate lyase gene (*Pcpel2*) from *Phytophthora capsici*. *Pcpel2 *was constructed by pET system and pMAL system, and both constructs were used to express the PCPEL2 in *Escherichia coli *BL21 (DE3) pLysS. The expressed products were purified using affinity chromatography and gel filtration chromatography. The purity, specific activity and pathogenicity of the purified PCPEL2 expressed by the pMAL system were higher than the purified PCPEL2 expressed by the pET system. In addition, some other characteristics of the purified PCPEL2 differed from the two systems, such as crystallographic features. Purified PCPEL2 expressed by the pMAL system was crystallized by the hanging-drop vapour-diffusion method at 289 K, and initial crystals were grown.

**Conclusion:**

The two different methods and comparison presented here would be highly valuable in obtaining an ideal enzyme for the downstream experiments, and supply an useful alternative to purify some insoluble recombinant proteins.

## Background

*Phytophthora capsici *is a phytopathogenic oomycete that causes severe disease in a wide variety of plant species. During infection, pathogens produce a diverse range of cell wall-degrading enzymes, including cellulases, hemicellulases and pectinases [1]. Pectinases can degrade pectin which is a major component of the primary cell wall and middle lamella of plants. According to the action mode, pectinase can be divided into three different types: polygalacturonase, pectin methyl esterase and pectate lyase (EC 4.2.2.2) [2].

Pectate lyases (PELs), which cleave glycosidic bonds of pectate or low methylated pectin by a trans-eliminative mechanism to yield unsaturated products, are widely distributed among microorganisms, such as *P. capsici*[3,4]. PELs are enzymes which specifically catalyze the cleavage of the α-1,4-glycosidic linkages in the unesterified polygalacturonic acid (PGA) regions of plant cell walls and produce an unsaturated bond between C4 and C5 of each d-galacturonic acid unit [5,6], resulting in tissue maceration and cell death due to osmotic fragility [7]. Polysaccharide lyases are classified into 15 different families, including five families of PELs identified as family number 1, 2, 3, 9, and 10 [8,9]. PELs are distinguished by both specificity for a glycosidic linkage and different optimal pH [10]. Most PELs are active in the pH range 8-11, and Ca^2+ ^is required for enzymatic activity [11,12], while some members depend on Co^2+^, Mn^2+^, and Ni^2+^[13].

Besides playing an important role in the infection process of various pathogens and fruit ripening, PELs have great commercial significance in industrial applications, such as extraction and clarification of fruit juices [14,15], maceration of vegetables, scoring of cotton fabric, and retting of flax [16]. There is a high interest to improve the quality and efficiency of the PELs for industrial applications.

Genes encoding PELs have been cloned from a variety of species, including *Erwinia chrysanthemi*[17], *Zinnia elegans*[18], *Meloidogyne javanica*[19], Strawberry [20], etc. Some have been further characterized by tertiary structure of the protein sequence and biochemical properties of the enzyme [21,22]. Most of the PELs were expressed as soluble recombinant proteins, such as PelW [23] and PelC [24] expressed in prokaryotic organism, and Pel1 [25] expressed in eukaryotic cells. A few recombinant proteins were insoluble [26], and the inclusion bodies required solubilization in 8 M urea.

Preparation of large-scale soluble protein can provide a basis for detailed structural and functional studies and ultimately improve our understanding of PEL. In the present study, we report the expression and purification of a new PEL from *P. capsici *using the pET and the pMAL systems. We identified important differences in the characteristics of the recombinant PELs produced by these two methods.

## Results and discussion

### Expression and purification of 6 × His-PCPEL2

**Figure 1:**
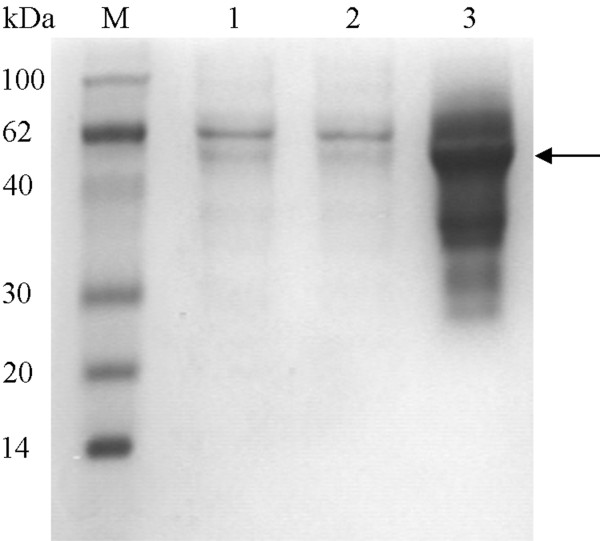
**Solubilization of PCPEL2 expressed by pET system**. Lane M, molecular mass marker; lanes 1 and 2, the supernatant of fusion protein after ultrasonication at pH 7.0 and 9.0 buffer (10 mM Tris-HCl, 150 mM NaCl); lane 3, the supernatant of fusion protein after ultrasonication with suspending buffer (10 mM Tris-HCl pH 10.3 containing 150 mM NaCl, 2 M urea, 10 mM imidazole and 5 mM β-mercaptoethanol). The over-expressed protein is indicated by the arrow.

**Figure 2:**
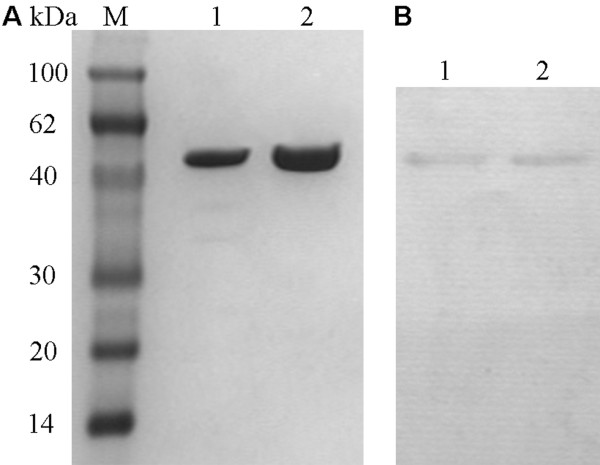
**Purification of PCPEL2 with the two systems**. (A) The purified PCPEL2. Lane M, molecular mass marker; lane 1, the purified PCPEL2 expressed by pET system; lane 2, the purified PCPEL2 expressed by pMAL system. (B) Confirmation of purified PCPEL2 by Western blot.

The mature fragment of *Pcpel2 *(1137 bp) without introns was obtained and cloned into a pET28a expression vector. The recombinant plasmid was transformed into *E. coli *BL21 (DE3) pLysS cells, following by induction under the T7 promoter with 1 mM IPTG for 20 h at 16°C. The predicted molecular mass of the recombinant protein 6 × His-PCPEL2 is 44 kDa. After being lysed by sonication, there was little target protein (6 × His-PCPEL2) in supernatant at pH 7.0-9.0 buffer (10 mM Tris-HCl, 150 mM NaCl) (Figure 1, Lanes 1 and 2). The insoluble particles were solubilized with buffer A (10 mM Tris-HCl pH 10.3 containing 150 mM NaCl, 2 M urea, 10 mM imidazole and 5 mM β-mercaptoethanol), and then most of the expressed recombinant proteins were soluble (Figure 1, Lane 3). Both the higher pH (pH 10.3) and the urea were essential for the solubilization of the recombinant protein 6 × His-PCPEL2. After a series of purification steps (Table 1), the purified protein was more than 90% purity by SDS-PAGE stained with Coomassie brilliant blue (Figure 2A, Lane 1). Analysis by SDS-PAGE showed a significant amount of a 44 kDa protein containing a 6 × His tag at the N-terminus of PCPEL2, in agreement with the predicted molecular weight for this protein. The protein was also recognized by western blot analysis (Figure 2B, Lane 1).

**Table 1:** Purification summary of PCPEL2 expressed by pET system

Purification steps	Total protein (mg)	Yield (%)	Total activity^b ^ (U)	Specific activity (U/mg protein)	Purification (fold)
Crude supernatant^a^	702.5	100	15455	22	1
Ni-NTA affinity chromatography	20.2	2.88	3131	155	7.0
Superdex-200	6.0	0.85	4500	750	34.1

^a ^The starting material was 1 L of crude *E. coli *supernatant.

^b ^Enzyme activity was defined as the amount of enzyme that produced a change in absorbance of 0.001 at 232 nm/min, as determined by the formation of unsaturated uronide.

### Expression and purification of MBP-PCPEL2

**Figure 3:**
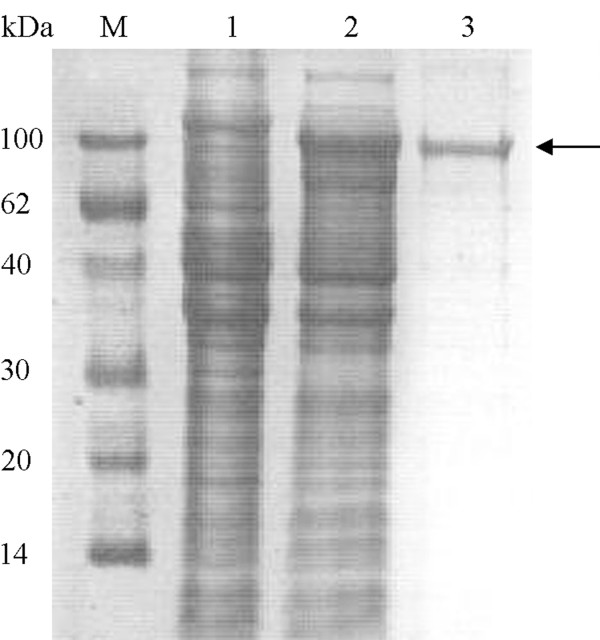
**Expression and purification of fusion protein expressed by pMAL system**. Lane M, molecular mass marker; lanes 1, un-induced bacterial lysate; lane 2, IPTG-induced culture; lane 3, product eluted from the Amylose Resin column (arrow indicates a 86 kDa purified fusion protein, corresponding to the 42 kDa MBP plus 44 kDa PCPEL2).

PCPEL2 was over-expressed as a soluble protein in *E. coli *using pMAL expression system. It is shown from SDS-PAGE analysis that cell lysate dominantly reveals a 86 kDa band (Figure 3, Lane 2), corresponding 42 kDa MBP plus 44 kDa PCPEL2. The expressed MBP-PCPEL2 comprised 40% of the total protein, most of which was soluble. The soluble fusion protein dissolved in buffer 1 (10 mM Tris-HCl pH 7.5 containing 150 mM NaCl and 5 mM β-mercaptoethanol) was firstly purified by Amylose Resin column based on MBP affinity (Figure 3, Lane 3). To remove the affinity tag from fusion protein MBP-PCPEL2, an enzymatic cleavage procedure was conducted with human rhinovirus 3C protease, a protease specific for a spacer sequence between MBP and PCPEL2. The result implies that the human rhinovirus 3C protease can efficiently cleave the corresponding tag fused to the target protein. To separate the PCPEL2 from the enzymatic mixture of MBP-PCPEL2, we tried a method of amylose re-affinity. The PCPEL2 purity was about 95% and showed a significant amount of a 44 kDa protein, as analyzed by SDS-PAGE (Figure 2A, Lane 2). The protein was further verified by western blot analysis (Figure 2B, Lane 2).

### *In vitro *and *in vivo *activity assay

**Figure 4:**
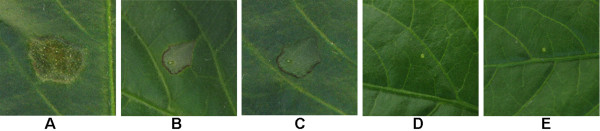
**Symptoms appearing on pepper leaves after inoculation treatments**. (A) Zoospore suspensions (positive control); (B) purified PCPEL2 expressed by pET system; (C) purified PCPEL2 expressed by pMAL system; (D) heat-inactivated protein (negative control); (E) distilled water (negative control).

The activity of the purified PCPEL2 was analyzed both *in vitro *and *in vivo*. The specific activity towards Polygalacturonic Acid of the purified PCPEL2 that was produced using the pET system and the pMAL system was 750 U/mg and 860 U/mg, respectively. In the *in vivo *activity assay, the presence of lesions in the positive control leaves (Figure 4A) was regarded as a typical disease symptom compared with the treated leaves. The treated leaves began to display small necrotic spots at the second day after treatment (dat) with PCPEL2, and the necrotic lesions expanded gradually and visible necrotic lesions appeared after 4 dat (Figure 4B and 4C). In addition, the mean lesion area treated with the purified PCPEL2 expressed by the pMAL system was significantly bigger (Figure 4C) than that of treated with the purified PCPEL2 expressed by the pET system, in agreement with the specific activities. PCPEL2 could induce necrotic lesions on the pepper leaves, resulting in pathogenic symptoms. The results confirm that PCPEL2 is likely to soften pepper tissues and may cause death of plant cells, and might be one of the pathogenicity factors during infection of *P. capsici*.

### Crystallization of PCPEL2

**Figure 5:**
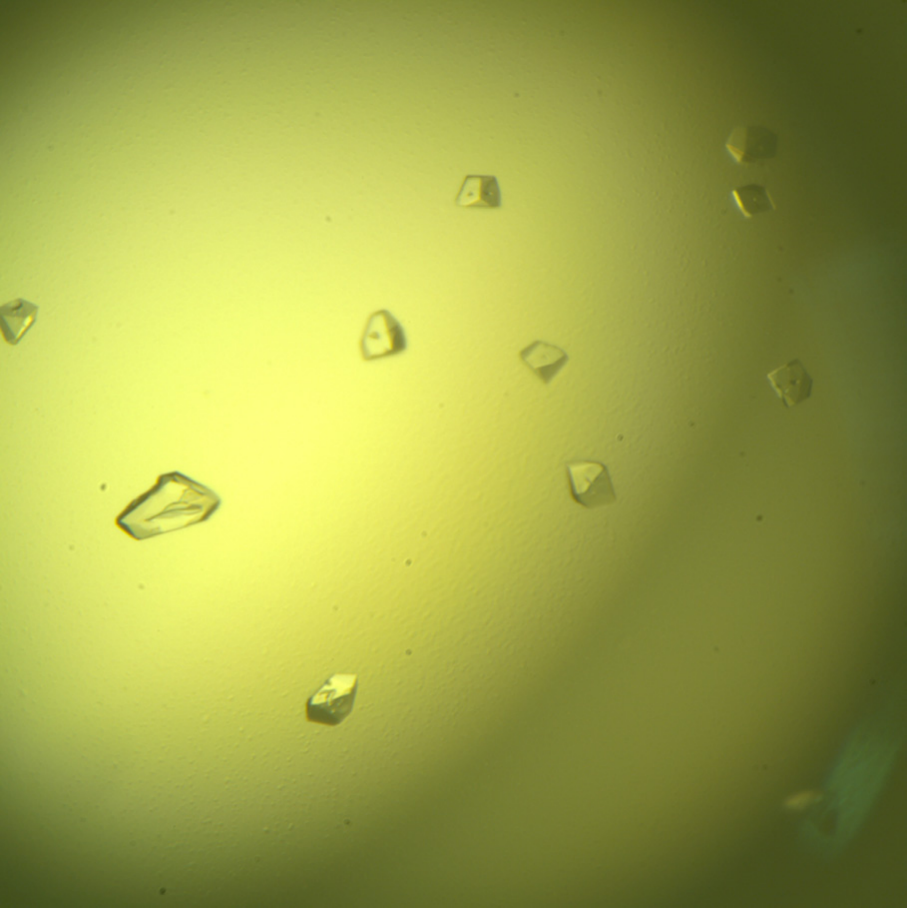
**Initial crystals of PCPEL2**.

The purified PCPEL2 obtained by the two different ways was used for the crystallization trials. Initial crystals of PCPEL2 (Figure 5) appeared using the purified protein expressed by the pMAL system. The lower pH and higher purity of the PCPEL2 produced by the pMAL system compared to the purified protein expressed by the pET system may have contributed to the good crystallization of the former PCPEL2. The crystals were grown in drops with 0.1 M HEPES (pH 7.5), 10% (w/v) Polyethylene glycol 6000, 5% (*v/v*) (±)-2-Methy-2,4-pentanediol. High quality crystals of PCPEL2 would be necessarily optimized in order to get a good X-ray diffraction data set for the structure determination. The work is in progress.

### Characterization comparison of the two purified PCPEL2

The buffer dissolved the PCPEL2 and the purity of the purified PCPEL2 differed from the two systems. As far as PCPEL2 expressed by the pET system, the higher pH (pH 10.3) and the urea (2 M) were essential during the purification steps, and the urea could be removed gradually by dialysis. The above steps were unnecessary for the PCPEL2 expressed by the pMAL system since the MBP tag can increase the solubility of target protein. Specific activities of purified PCPEL2 compared favorably at 750 and 860 U/mg protein, respectively (Tables 1 and 2). In addition, both the two purified PCPEL2 were able to induce necrotic lesions on the pepper leaves, but the necrotic lesions were different from the typical Phytophthora foliar blight symptoms as observed in the positive control. The different symptoms are possibly because *P. capsici *is likely to be producing a series of pathogenesis-related enzymes to initiate host cell death, while the PCPEL2 is a single pathogenicity factor to soften pepper tissues. The two purified PCPEL2 showed differences in infectious characteristic, the mean lesion area treated with the higher activity PCPEL2 was distinctly bigger than that of treated with the lower activity protein, suggesting the virulence of PCPEL2 enzyme is related to its activity. Crystals of PCPEL2 were grown using the purified protein expressed by the pMAL system, while no crystals appeared using the purified PCPEL2 expressed by the pET system. In conclusion, the purified PCPEL2 expressed by the pMAL system contributed to the higher specific activity, pathogenicity and crystallization.

**Table 2:** Purification summary of PCPEL2 expressed by pMAL system

Purification steps	Total protein (mg)	Yield (%)	Total activity^b ^ (U)	Specific activity (U/mg protein)	Purification (fold)
Crude supernatant^a^	860.5	100	13768	16	1
Amylose Resin affinity chromatography with MBP	22.6	2.63	3051	135	8.4
Amylose Resin affinity chromatography without MBP	7.5	0.87	6450	860	53.8

^a ^The starting material was 1 L of crude *E. coli *supernatant.

^b ^Enzyme activity was defined as the amount of enzyme that produced a change in absorbance of 0.001 at 232 nm/min, as determined by the formation of unsaturated uronide.

## Conclusions

In summary, we cloned a new pectate lyase gene (*Pcpel2*) from *P. capsici*. PCPEL2 was expressed by two different systems, pET and pMAL systems. The recombinant products were purified using affinity chromatography and gel filtration chromatography. The purity, specific activity and pathogenicity of the purified PCPEL2 expressed by the pMAL system were higher than the purified PCPEL2 expressed by the pET system. In addition, some other characteristics of the purified PCPEL2 differed from the two systems, such as crystallographic features. Using different methods and comparison of the products would be highly valuable in obtaining an ideal enzyme for the downstream experiments.

## Methods

### Strains, plasmids and chemicals

The pectate lyase gene *Pcpel2 *[GenBank Accession No. FJ213435] was obtained by screening the genomic library of *P. capsici *by our lab [27]. DH5α Chemically Competent Cell (TransGen) was used as a host strain for manipulation. *E. coli *BL21 (DE3) pLysS (TransGen) was used as expression strain. The pET28a Expression kit was obtained from Novagen. The pET28a vector contains the T7 promoter and allows expression of the recombinant protein fused to His-tag at the N-terminus. Vector pMAL-p4X, purchased from New England Biolabs, was reconstructed by our lab. The human rhinovirus 3C protease was expressed and purified as previously described [28]. Restriction enzymes, T4 DNA ligase, *Pfu *polymerase and protein marker were purchased from TaKaRa (Dalian, China). All chemicals were obtained from Sigma-Aldrich unless otherwise specified.

### Construction of plasmids pET28-Pcpel2 and pMAL-Pcpel2

A signal peptide of 22 amino acids of *Pcpel2 *was predicted by the SignalP program [29]. The N-terminal signal peptide sequence was omitted. The mature fragment of *Pcpel2 *was amplified by PCR using sense 5'-CGCGGATCCCTCTCCACGGGTACGGCACCAG-3' and antisense 5'-CCGGAATTCTTAGTTGGACAGGACACCCAC-3' primers (Invitrogen), containing BamHI and EcoRI restriction sites (underlined), respectively. After initial denaturation at 95°C for 2 min, the PCR reaction was carried out using *TransStart FastPfu *DNA Polymerase (TransGen). The conditions for each cycle were as follows: denaturation at 95°C for 20 s, annealing at 63°C for 30 s, and extension at 72°C for 20 s. A final extension step at 72°C for 5 min was added at the end of the 35 cycles. The purified PCR product was digested with BamHI and EcoRI, and ligated into the BamHI-EcoRI site of the expression vectors pET28a and reconstructed pMAL-p4X, generating pET28-Pcpel2 and pMAL-Pcpel2 plasmids. The recombinant plasmids were transformed into *E. coli *BL21 (DE3) pLysS cells for inducible expression. The recombinant plasmid pET28-Pcpel2 has a 6 × His tag at the N-terminus, while the recombinant plasmid pMAL-Pcpel2 has a *malE *gene, which encodes maltose-binding protein (MBP).

### Expression and purification of 6 × His-PCPEL2

Cells carrying the recombinant 6 × His-PCPEL2 were grown at 37°C in LB medium containing 50 ug/ml kanomycin until they reached an optical density at 600 nm of 0.5, and then the cells were induced with 1 mM IPTG for 20 h at 16°C. Cells were harvested, resuspended in buffer A (10 mM Tris-HCl pH 10.3 containing 150 mM NaCl, 2 M urea, 10 mM imidazole and 5 mM β-mercaptoethanol) and lysed by sonication. The lysate was centrifuged at 15,000 g for 30 min at 4°C, and the supernatant was loaded onto a Ni-NTA Superflow column (GE Healthcare) equilibrated with buffer A. The column was washed with buffer B (buffer A containing 20 mM instead of 10 mM imidazole), buffer C (buffer B containing 0.5% (*v/v*) tween-20) and buffer D (buffer B containing 0.5% (*v/v*) ethanol). The proteins were eluted with buffer E (buffer A containing 100 mM instead of 10 mM imidazole). The solution was concentrated in a centrifugal concentrator (Millipore) and applied onto a Superdex-200 gel-filtration column equilibrated in 10 mM Tris-HCl pH 10.3, 150 mM NaCl, 2 M urea and 1 mM DTT. Fractions were collected according to their UV absorption peaks. Major peak fractions containing PCPEL2 were pooled and dialyzed overnight against buffer F (10 mM Tris-HCl pH 10.3 containing 150 mM NaCl and 1 mM DTT). The sample was then concentrated to 2 mg/ml according to the Bradford assay. The molecular mass and the purification steps of PCPEL2 were determined using SDS-PAGE (12% polyacrylamide).

### Expression and purification of MBP-PCPEL2

The conditions of the fermentation and induction with the cells carrying the recombinant MBP-PCPEL2 were the same as those of the recombinant 6 × His-PCPEL2 described above. Cells were harvested, resuspended in buffer 1 (10 mM Tris-HCl pH 7.5 containing 150 mM NaCl and 5 mM β-mercaptoethanol) and lysed by sonication. The lysate was centrifuged at 15,000 g for 30 min at 4°C, the supernatant was loaded onto an Amylose Resin column (New England Biolabs) which pre-equilibrated with buffer 1. To remove non-specifically bound proteins, the column was washed with buffer 1, buffer 2 (buffer 1 containing 0.5% (*v/v*) tween-20) and buffer 3 (buffer 1 containing 1% (*v/v*) triton X-100). The fusion protein was eluted with buffer 4 (10 mM Tris-HCl pH 7.5 containing 150 mM NaCl, 1 mM DTT and 10 mM maltose). The purity of the MBP-PCPEL2 was determined by SDS-PAGE. To separate MBP from PCPEL2, the purified fusion protein was digested with human rhinovirus 3C protease at the weight ratio of 50:1 (substrate/enzyme). The released enzyme was purified with an Amylose Resin column to retain MBP protein.

### Western blot analysis

The purified PCPEL2 was used to prepare antibody in New Zealand white rabbits [30]. The antibody preparation, purification and antiserum titer determination were done as previously described [31]. The proteins purified by the two different ways were transferred to a polyvinylidene fluoride (PVDF) membrane after SDS-PAGE. The membrane was blocked with 5% (*w/v*) non-fat dry milk diluted in TBS buffer (10 mM Tris-HCl, pH 7.0, 100 mM NaCl) for 1 h, and then rinsed with TBS and placed in 1:500 antibody for 2 h by constant shaking. The membrane was then washed with washing buffer (TBS buffer containing 0.5% (*v/v*) tween-20) three times (5 min each) and incubated with 1:1000 diluted HRP-labeled goat anti-rabbit IgG (Glostrup, Denmark) in TBS buffer for 1 h. Immune complexes were detected by enhanced chemiluminescence according to the manufacturer's specifications (Santa Cruz, CA, USA).

### Enzyme activity assay

The purified PCPEL2 was used to determine the pectate lyase activity by adding 20 μl (2 mg/ml) protein solution to 980 μl of reaction buffer (0.2% Polygalacturonic Acid, 100 mM Tris-HCl, pH 8.5, and 1 mM CaCl_2_) and kept at 40°C for 10 min. Then, 250 μl of 50 mM HCl was added to stop the enzymatic reaction and absorbance at 232 nm was measured with a U-2100 spectrophotometer (Amersham Biosciences). The enzymatic activity was defined as the amount of enzyme that produced a change in absorbance of 0.001 at 232 nm/min [32], as determined by the formation of unsaturated uronide [33].

### Treatment of pepper leaves with PCPEL2

Prior to inoculation, seedlings of Pepper Kexing 3 were maintained at 100% humidity at 28°C for 24 h to maximize leaf opening during the fifth to sixth-leaf stage. To evaluate the impact of the purified PCPEL2 on leaves, the seedlings were spot inoculated with 2.5 μl (2 mg/ml) protein solution using a microsyringe. Inoculations were carried out on three of the uppermost leaves. A second set of leaves inoculated with zoospore suspensions (1 × 10^5^/ml) served as a positive control while the heat-inactivated protein and distilled water served as negative controls. Following inoculation, seedlings were maintained at 100% humidity at 28°C for 7 days.

### Crystallization of PCPEL2

The purified PCPEL2 was concentrated to 5 mg/ml in 10 mM Tris-HCl, pH 7.5, 150 mM NaCl and 1 mM DTT. The Crystal Screen I and Screen II (Hampton Research) sets of screening conditions were used for the first screening. Initial crystallization conditions were established using 16-well Linbro plates. Purified PCPEL2 was initially crystallized using the hanging-drop vapour-diffusion method at 289 K with 1 μl protein solution and 1 μl mother liquor and the mixture was equilibrated against 200 μl reservoir solution, following by a refinement of the conditions through the variation of precipitants, pH, protein concentrations and additives. Initial crystals appeared after 5 days.

## Competing interests

The authors declare that they have no competing interests.

## Authors' contributions

XGZ conceived and designed the experiment. HZW and LF performed the experiments. HZW analyzed the data. HZW and XGZ wrote the paper. All authors read and approved the final manuscript.
